# Screening and identification of cyprinid herpesvirus 2 (CyHV-2) ORF55-interacting proteins by phage display

**DOI:** 10.1186/s12985-023-02026-x

**Published:** 2023-04-12

**Authors:** Min Qian, Simin Xiao, Yapeng Yang, Fei Yu, Jinxuan Wen, Liqun Lu, Hao Wang

**Affiliations:** 1 National Pathogen Collection Center for Aquatic Animals, Shanghai Ocean University, Shanghai, 201306 China; 2 Institute of Marine Biology, College of Oceanography, Hohai University, Nanjing, 210098 China; 3 National Demonstration Center for Experimental Fisheries Science Education, Shanghai Ocean University, Shanghai, 201306 China; 4 Key Laboratory of Freshwater Aquatic Genetic Resources, Ministry of Agriculture, Shanghai Ocean University, Shanghai, 201306 China

**Keywords:** Cyprinid herpesvirus 2, ORF55, Prokaryotic expression, Phage display technology, Thymidine kinase, Virulence factor

## Abstract

**Background:**

Cyprinid herpesvirus 2 (CyHV-2) is a pathogenic fish virus belonging to family Alloherpesviridae. The CyHV-2 gene encoding thymidine kinase (TK) is an important virulence-associated factor. Therefore, we aimed to investigate the biological function of open reading frame 55 (ORF55) in viral replication.

**Methods:**

Purified CyHV-2 ORF55 protein was obtained by prokaryotic expression, and the interacting peptide was screened out using phage display. Host interacting proteins were then predicted and validated.

**Results:**

ORF55 was efficiently expressed in the prokaryotic expression system. Protein and peptide interaction prediction and dot-blot overlay assay confirmed that peptides identified by phage display could interact with the ORF55 protein. Comparing the peptides to the National Center for Biotechnology Information database revealed four potential interacting proteins. Reverse transcription quantitative PCR results demonstrated high expression of an actin-binding Rho-activating protein in the latter stages of virus-infected cells, and molecular docking, cell transfection and coimmunoprecipitation experiments confirmed that it interacted with the ORF55 protein.

**Conclusion:**

During viral infection, the ORF55 protein exerts its biological function through interactions with host proteins. The specific mechanisms remain to be further explored.

**Supplementary Information:**

The online version contains supplementary material available at 10.1186/s12985-023-02026-x.

## Introduction

*Herpesviral haematopoietic necrosis* (HVHN), a highly lethal disease of crucian carp (*Carassius carassius*), is caused by cyprinid herpesvirus 2 (CyHV-2). This disease was first detected in goldfish (*Carassius auratus*) cultured in Aichi and Nara prefectures in Japan in 1992, with a mortality rate of 100% [1]. Since 2009, CyHV-2 has been detected in Gibel Carp (*Carassius auratus gibelio*) cultured in some areas of Jiangsu Province in China, and the virus rapidly became endemic throughout the main carp farming areas in China [2]. CyHV-2 has since dramatically spread across the world [3–6]. CyHV-2 is a member of the *Alloherpesviridae* family (genus *Cyprinivirus*), which also comprises cyprinid herpesvirus 3 (CyHV-3) and carp pox virus (CyHV-1) [7]. CyHV-2 is a DNA virus with a vesicular membrane and a large linear double-stranded DNA genome. The virions are oval, with a diameter of approximately 170 − 200 nm [8].

Developing the molecular epidemiological characterisation of CyHV-2 is an important basis for research on the infection mechanism, and for prevention and control of the virus. Some genes of CyHV-2 have been used for research on virus detection, preparation of antibody, and genotype distinction and diversity analysis [9–11]. Additionally, the CyHV-2 open reading frame 55 (ORF55) gene was predicted to encode thymidine kinase (TK), and enzyme participating in nucleic acid metabolism [12]. In previous studies, the function of TK was investigated in herpes simplex virus 1 (HSV-1) [13–15], duck plague virus (DPV) [16] and Epstein-Barr (EB) virus [17]. The TK gene has since been studied in Pseudorabies virus (PRV) [18]. The TK gene was not essential for the growth of PRV, but it was essential for viral proliferation, and it was also the most important gene determining PRV critical virulence. Additionally, deletion of the TK gene could significantly decrease the virulence of PRV, while also greatly reducing the replication, transmission and lethal ability of the virus in nerve tissues, but immunogenicity was not affected [19].

Thus, TK plays an important role during viral infection. Although the experimental evidence mentioned above linked the TK gene with virulence, its overall specific mechanism of action remains elusive. From previous experimental results, we deduced that ORF55 also has a virulence effect, and is an important virulence gene in CyHV-2. Therefore, further exploration of the ORF55 gene is important.

Phage display technology (PDT) is a powerful approach first reported in 1985 [20]. PDT is a well-established method for selecting peptides and proteins with novel binding functions [21]. A foreign protein is first expressed in fusion with one of the phage capsid proteins by cloning the foreign DNA sequence in frame with the gene encoding the chosen capsid protein [22]. PDT allows a direct link and identification between a large number of random peptides and DNA-coding sequences. It is used extensively in medicine, biology and chemistry [23, 24]. However, how the CyHV-2 ORF55 protein performs its biological functions in goldfish cells remains poorly understood. Screening and identifying peptides or proteins that may interact with the ORF55 protein could help to elucidate its mechanism of action.

In this study, we cloned the ORF55 gene into a prokaryotic expression vector using seamless cloning, and used PDT to screen interacting partners. Bioinformatics prediction identified four potential interacting proteins. This study lays a foundation for further exploration of the biological functions of the ORF55 protein during viral infection.

## Materials and methods

### Bacterial strains, cloning vectors, cells and virus

*Escherichia coli DH5α* and *E. coli BL21* (*DE3*) strains were obtained from TransGen Biotech (Beijing, China). The prokaryotic expression vector *pMAL-c5X* was purchased from New England Biolabs (NEB; Beijing, China). The CyHV-2 YC-01 strain (GenBank accession No. MN593216; 275,367 bp) was isolated and purified from Crucian Carp showing obvious symptoms of the early stages gill haemorrhage in the laboratory at Shanghai Ocean University, and preserved at − 80 °C. A cell line from the caudal fin of *C. auratus gibelio* (GiCF) [25] was established in previous experiments and cultured in M199 (Genomcell, Zhejiang, China) medium supplemented with 12% fetal bovine serum (FBS; Every Green, Zhejiang, China) at 27 °C. This cell line could be stably infected with CyHV-2 and used for in vitro virus replication. HEK-293 T cells were cultured in Dulbecco’s modified Eagle’s medium with high glucose containing 10% FBS in a temperature-controlled incubator at 37 °C with 5% CO_2_.

### Sequence-based prediction

The National Center for Biotechnology Information (NCBI) database of the National Library of Medicine was used to obtain the sequence of the TK gene from the CyHV-2 YC-01 strain. The ExPASy ProtParam tool (https://web.expasy.org/protparam/), a server that calculates theoretical protein parameters including molecular weight, amino acids, isoelectric point (pI) and instability index, was used assess the protein physical and chemical properties [26]. In order to explore the functions of the ORF55 protein, we applied different in silico approaches using Cell-PLoc (http://www.csbio.sjtu.edu.cn/bioinf/Cell-PLoc/), PSORTb (https://www.psort.org/psortb/), lncLocator (http://www.csbio.sjtu.edu.cn/bioinf/lncLocator/), iLoc-LncRNA (http://lin-group.cn/server/iLoc-LncRNA/home.php) and Euk-mPLOC (http://www.csbio.sjtu.edu.cn/bioinf/euk-multi-2/) to predict localisation and other characteristics [27–31]. The secondary structure of the ORF55 protein sequence was predicted by various web servers including SOPMA (https://npsa-pbil.ibcp.fr/cgi-bin/npsa_automat.pl?page=npsa_sopma.html), NovoPro (https://novopro.cn/tools/secondary-structure-prediction.html), GOR (http://gor.bb.iastate.edu/) and PSSpred (https://zhanggroup.org/PSSpred/) [32–35]. Also, we downloaded the TK gene sequences of other species and performed evolutionary tree analysis using MEGA version 7.0. For homology analysis as well as sequence alignment, TK genes of all published CyHV-2 strains and representative CyHV-1 and CyHV-3 strains were selected for comparison. Protein tertiary structure was predicted using I-TASSER (https://zhanggroup.org/I-TASSER/).

### Seamless cloning

The ORF55 gene was amplified using PCR with sense and antisense primers 5′-GCGATATCGTCGACGGATCCATGGCTTTTCTGGAGTTGGTGCTGG-3′ and 5′-CCTGCAGGGAATTCGGATCCTCATACAAAAGAACAAGGGGCATCC-3′. Restrictions sites for *Bam*HI were inserted in both primes. Thermal cycling included an initial denaturation step at 98 °C for 5 min, followed by 34 cycles of denaturation at 94 °C for 30 s, annealing at 58 °C for 30 s, and extension at 72 °C for 30 s, and a final extension at 72 °C for 5 min. The subsequent ligation reaction was carried out at 50 °C according to the *pEASY*-Basic Seamless Cloning and Assembly Kit (TransGen Biotech), after which ligation products were transformed into *E. coli DH5α*. Positive colonies were screened and plasmid was isolated and sent to Sangon (Shanghai, China) for DNA sequencing.

### Protein purification

Transformed *E. coli* BL-21 (DE3) cells were grown in Luria Bertani (LB) broth media containing 100 µg/mL ampicillin, and isopropylthiogalactoside (IPTG) was added to induce protein expression. Cells were collected by centrifugation and resuspended in 40 mL of 30 mM Tris–HCl, 20% sucrose, 1 mM EDTA, and mixed with 40 mL MgSO_4_. Following complete ultrasonic lysis and clarification by centrifugation, the bacterial supernatant was incubated with Amylose Resin (Sangon) on ice and transferred to an affinity chromatography column. After column washing, protein was eluted in elution buffer (20 mM Tris–HCl, 0.2 mol/L NaCl and 1 mM EDTA) containing 10 mM maltose.

### Western blotting

The bacterial solution before and after induction, as well as the purified protein, were separated by 12% SDS-PAGE at 80 V for 30 min followed by 120 V for 60 min. Gels were stained with Coomassie Brilliant Blue. For western blotting, purified protein was subjected to electroblotting on a polyvinylidene difluoride (PVDF) membrane, which was subsequently blocked with 5% nonfat milk. The membrane was incubated with anti-MBP-Tag monoclonal antibody as primary antibody (Proteintech, USA) then with goat anti-mouse IgG (H + L)-horseradish peroxidase (HRP) conjugate (Bioworld Technology, USA) as secondary antibody. Results were detected using an enhanced chemiluminescent (ECL) detection system.

### Phage display

The ORF55 protein concentration was determined from a standard curve prepared using bovine serum albumin (Sangon) and a Bicinchoninic Acid (BCA) Protein Quantification Kit (Beijing Solarbio Science & Technology Co. Ltd., Beijing, China). Protein was seeded in a 12-well plate at a concentration of 100 μg/mL, with three replicates for each experimental group. The phage library (NEB) was bound to the ORF55 protein according to the instruction manual of the Random Ph.D.-12 Phage Display Peptide Library Kit. Following three rounds of panning, the plate with plaques was sent to Genewiz Biotechnology Co. Ltd. (Tianjin, China) for sequencing and identification. The plaque sequencing were compared with NCBI crucian carp and goldfish databases.

### Interaction validation

As described above, we obtained polypeptides that may interact with the ORF55 protein through PDT. The molecular docking tool from Home for researchers was used to predict interactions between the ORF55 protein and polypeptides, and to confirm their specific interaction sites. Dot blot experiments were also performed to verify interactions. Serially diluted peptides were blotted onto a PVDF membrane, soaked in ORF55 protein solution, and successively incubated with primary and secondary antibodies as described above for western blotting.

### Protein prediction

Sequences of the four potential interacting proteins were downloaded from NCBI databases, and their sequences in other hosts were obtained for alignment, tertiary structure prediction, evolutionary tree analysis, and prediction of other physicochemical properties.

### Preliminary in vitro validation

GiCF cells were infected with the supernatant of CyHV-2 YC-01 and sampled at various timepoints to observe whether the virus solution could alter expression of the above genes. RNA was extracted from sampled cells using TRIzol reagent and reverse-transcribed to cDNA as a template for gene amplification. Gene expression was measured using PrimeScript RT Master Mix (Takara, Beijing, China) from amplified cDNA. A real-time system instrument was used to quantify gene expression levels. Reactions contained 6.5 µL of TB Green Premix Ex Taq II (2 × ; Takara), 0.5 µL forward/reverse primers, and 500 ng cDNA. Reactions were repeated in triplicate.

### Subcellular localisation analysis

Based on the above results, two proteins most likely to interact with the ORF55 protein were selected for further validation. Eukaryotic expression plasmids were constructed using seamless cloning technology as described above. Two luciferase reporter genes were selected as vectors. Unlike prokaryotic expression, a free endotoxin plasmid extraction kit was required for amplification. Recombinant plasmids were co-transfected into HEK-293 T cells. After 40 h, cell nuclei were stained using DAPI, and photographed using a confocal microscope.

### Coimmunoprecipitation analysis

Samples of cells co-transfected as described above were collected to determine whether proteins were successfully expressed. Cells were lysed using NP40 lysate, and green fluorescent protein (GFP) fusion-tagged proteins and their interacting partners were selected by rabbit anti-GFP immunomagnetic beads. Western blotting was performed to probe interactions between proteins using anti-RFP Tag mouse monoclonal antibody as primary antibody as described above.

### Statistical analysis

Statistical analysis was performed using GraphPad Prism (https://www.graphpad.com/scientific-software/prism/) and IBM SPASS statistics (SPASS 19.0). Results are presented as mean ± standard error (SE). Statistical significance was calculated using one-way analysis of variance (ANOVA). Values were considered significant (*) for *p*-values of 0.01 to 0.05, very significant (**) for *p*-values of 0.001 to 0.01, and extremely significant (***) for *p*-values 0 to 0.001.

## Results

### Physiochemical parameters

The physicochemical properties of the CyHV-2 YC-01 ORF55 protein were predicted using the Protparam web tool (Table 1). The predicted pI of ORF55 was < 7, suggesting it is acidic. The instability index value indicated that it is an unstable protein in CyHV-2 and CyHV-3, but stable in CyHV-1. The calculated aliphatic index indicated that it was thermally over a wide temperature range. ORF55 was classified as polar based on the grand average hydropathy (GRAVY) value. Knowing where the ORF55 protein is localised is critical to predicting its functions. All different algorithm-based servers predicted that the ORF55 protein is in the cytoplasm (Table 2). Almost all servers predicted that the ORF55 protein is rich in the alpha-helix secondary structure, which is likely important for protein structure and function (Table 3).

**Table 1:** Physiochemical profile of ORF55 protein in different virus strain type

Virus strain	AA	MW	pI	EC	Instability Index	Aliphatic Index	GRAVY
SY-C1	211	23.48	4.54	14,940	40.91 Unstable	84.08	0.017
YC-01	214	23.86	4.44	14,940	42.90 Unstable	82.90	− 0.032
CyHV-1 NG-J1	181	19.79	6.30	7950	24.69 Stable	108.29	0.318
CyHV-3 T	224	24.62	6.31	18,380	44.11 Unstable	84.91	0.030

The ORF55 protein’ amino acid sequences from different virus strains were all obtained from NCBI database. The physicochemical properties for this protein was calculated using the Protparam web service

**Table 2:** Subcellular localization prediction of ORF55 protein

S.No	Webserver	Predicted localization
1	Cell-PLoc 2.0	Cytoplasm & Nucleus
2	PSORTb	Cytoplasm
3	lncLocator	Cytoplasm & Nucleus
4	iLoc-LncRNA	Cytoplasm
5	Euk-mPLOC	Cytoplasm

Knowing where ORF55 protein is situated enables a more accurate prediction of its function. Using different software can get more accurate localization prediction

**Table 3:** Secondary structure profile of Rv1636 protein by the different webserver

Protein	Webservers	Alpha Helix	Extended Strand	Random Coil
ORF55	SOPMA	87 (40.65%)	36 (16.82%)	76 (35.51%)
	NovoPro	94 (43.93%)	30 (14.02%)	90 (42.05%)
	GOR	72 (33.64%)	45 (21.03%)	52 (24.30%)
	PSSpred	74 (34.58%)	60 (28.04%)	80 (37.38%)

Various online servers were used for the two-dimensional (2D) structure prediction of the ORF55 protein

### Bioinformatics analysis of the ORF55 protein

**Fig. 1:**
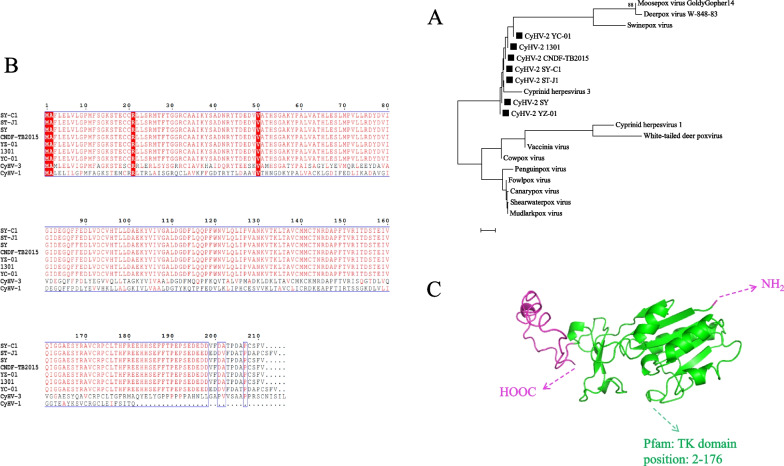
Bioinformatics analysis of the CyHV-2 ORF55 gene. **A** Evolutionary tree. Trees were generated with the neighbour-joining method using MEGA version 7. TK genes from different viruses were selected for simultaneous evolutionary tree generation, and TK from CyHV-2 is marked with a black square. Bootstrap values (1000 replicates) below 70% are hidden. **B** Amino acid sequences of TKs CyHV-1, CyHV-2 and CyHV-3. **C** Tertiary structure prediction. The tertiary structure of the ORF55 protein was generated by I-TASSER

A phylogenetic tree of TKs from different viruses was constructed using the neighbour-joining method in MEGA 7.0 software, revealing the evolutionary journey and relatedness of TK genes (Fig. 1A). Homology analysis in MegAlign 7.1 showed that all CyHV-2 strain types shared 100% sequence identity and > 50% identity with CyHV-1 and CyHV-3 (Additional file 2: Fig. 1G). All CyHV-2 strain types shared 98.6% similarity (Fig. 1B). Among them, ST-J1 and YC-01 strains were completely identical, with nine more bases (GGACGAGGA) than other strains. The results indicate that the ORF55 gene is highly conserved between CyHV-2 strains. The protein tertiary structure analysis (Fig. 1C) showed that there was a Pfam: TK domain located at 2–176, which laid a foundation for subsequent in-depth analysis of the reciprocal binding sites.

### Validation of protein expression

**Fig. 2:**
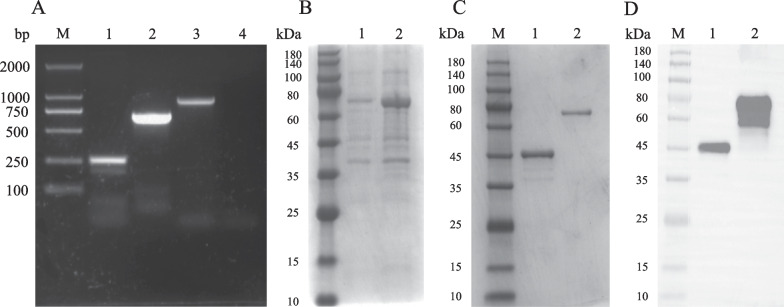
PCR amplification, prokaryotic expression, and validation of the ORF55 gene. **A** DNA extracted from the virus supernatant of infected cells was used as the amplification template, and the ORF55 gene was amplified by PCR with specific primers. M, DNA Marker 2000; 1, MBP gene (partial); 2, ORF55 gene; 3, MBP-ORF55 gene; 4, negative control. **B** Induced expression analysis of the ORF55 protein. The bacterial solution before and after IPTG induction was collected by centrifugation and analysed by SDS-PAGE. M, Protein markers; 1, uninduced; 2, induced. **C** SDS-PAGE analysis. The recombinant ORF55 protein was purified and the collected fractions were subjected to SDS-PAGE. The target protein was purified to a high concentration, and molecular weights of 45 kDa and 70 kDa were observed for bands corresponding to the MBP tag and ORF55 recombinant protein, respectively. M, Protein markers; 1, MBP protein; 2, ORF55 recombinant protein. **D** Western blotting analysis of purified ORF55 recombinant protein. A single band was observed. M, Protein markers; 1, MBP protein; 2, ORF55 recombinant protein

PCR results showed that the ORF55 gene was 645 bp in length, and 904 bp after connecting the prokaryotic expression vector, as expected (Fig. 2A). Expression of the ORF55 recombinant plasmid resulted in protein expression in *E. coli* BL-21 (DE3) following induction with 0.5 mM IPTG (Fig. 2B). SDS-PAGE results revealed a single band corresponding to MBP-tagged recombinant ORF55 protein, with molecular weights of 45 kDa for MBP and 70 kDa for ORF55 (Fig. 2C). Western blotting displayed a single band for purified recombinant ORF55 protein (Fig. 2D).

### Screening of interacting polypeptides

**Fig. 3:**
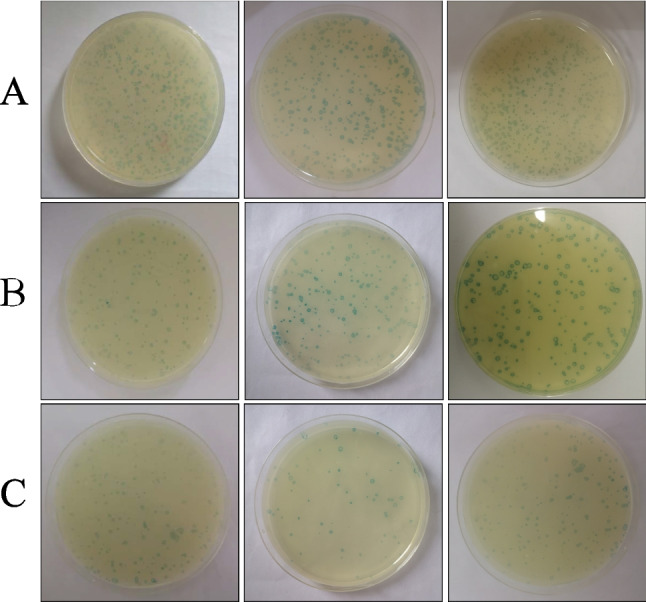
Phage plaque plating. Determining the phage enrichment for specific binding to the ORF55 protein for each round of screening requires a quantifiable indicator, hence visual counting and observation of phage plaques were performed for all three rounds of phage panning. Each panning was repeated three times. **A** First round of screening. **B** Second round of screening. **C** Third round of screening

A standard curve was established using BSA and the ORF55 protein concentration was measured at 562 nm using a spectrophotometer (Additional file 2: Fig. 2). During the three rounds of phage screening, the screening conditions were optimised by gradually reducing the amount of coated protein, increasing the concentration of Tween-20 and washing with detergent, and reducing the incubation duration with phage to obtain positive clones with high affinity (Table 4A). To determine phage enrichment for specific binding to the ORF55 protein in each round of screening requires a quantifiable indicator, in addition to the visual counting of phage plaques obtained in each round of phage panning (Fig. 3), hence phage titres after each round of screening were measured. The results showed that the correlation between the number of selection rounds and the titres of phages bound to ORF55 protein was positive (Table 4B). The recovery yield of phages was increased approximately 84-fold after the third round of panning compared with the first round. This indicates an obvious enrichment in the specific binding capability of phages.

**Table 4:** Enrichment, titer analysis and polypeptide screening of phages

Panning rounds	Target protein (µg/mL)	Tween-20 (V/V)	Washing times	Incubation time (min)
(A). Screening conditions for three rounds of panning				
First round	100	0.1%	10	80
Second round	90	0.2%	12	60
Third round	85	0.5%	15	50

Panning rounds	Input phage	Recovered phage	Recovery
(B). Recovery of Ph.D.-12 library screening			
First round	1.5 × 10^12^	2.9 × 10^4^	1.9 × 10^–8^
Second round	1.8 × 10^11^	1.3 × 10^5^	7.2 × 10^–7^
Third round	3 × 10^10^	1.5 × 10^5^	5 × 10^–6^

Nucleotide sequence	Repetition number	Translation
(C). Sequence analysis of specific binding peptide of ORF55 protein		
CTTTCGCCTGGGGCTAATAGTCATGTTTCTCGGCAT	38	LSPGANSHVSRH

Protein name	Number of amino acids
(D). Prediction of interactional host genes	
Gastrula zinc finger protein XlCGF8.2DB-like (ZFP)	371
WD repeat-containing protein 7 isoform X1 (WDR7)	1466
Actin-binding Rho-activating protein (ABRA)	157
Zinc finger protein 516-like Isoform X1 (ZFP516)	1065

**A** Screening conditions for three rounds of panning. During the three rounds of screening, the screening conditions were optimized by gradually reducing the amount of coated protein, increasing the concentration of Tween-20 and washing times, and reducing the incubation time with the phage to obtain positive clones with high affinity. **B** Recovery of Ph.D.-12 library screening. Phage titers were detected after each round of screening according to the manual M13 method. In each round of screening, input phage and recovered phage data were recorded, and recovery rate (%) was used to reflect the enrichment of specifically bound phages. **C** Sequence analysis of specific binding peptide of ORF55 protein. Fifty phage spots obtained in round 3 screening were randomly selected for DNA sequence determination. After translating the phage nucleic acid sequence into amino acids, the polypeptide sequence LSPGANSHVSRH had 38 repeats. **D** Prediction of Interactional host genes. The above polypeptide sequence and protein sequence alignment with the crucian carp library by NCBI were predicted to potentially interact with four crucian carp-related proteins.

### Prediction of interacting proteins

After the third round of screening, 50 plaque samples were randomly selected and sent to Genewiz for DNA sequencing. Based on the resulting data, nucleotide sequences were translated into amino acid sequences using DNAMAN (version 6). The results showed that the polypeptide sequence LSPGANSHVSRH had the highest homology, with 38 repetitions (Table 4C), suggesting it binds strongly to the ORF55 protein.

### Validation of polypeptide-protein interactions

**Fig. 4:**
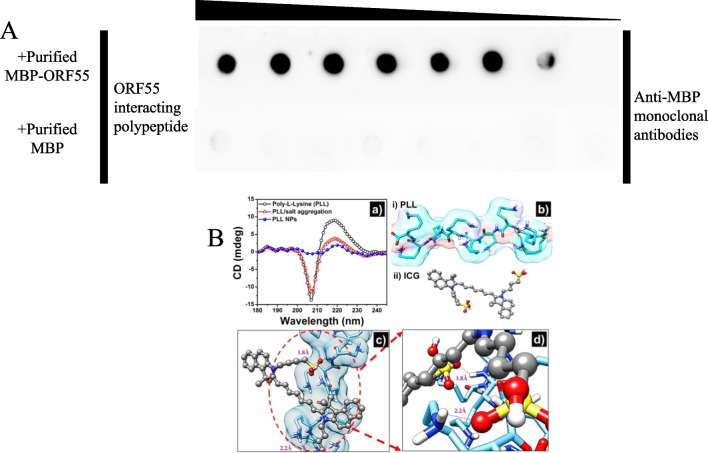
Prediction and verification of the interaction between ORF55 protein and polypeptide. **A** Dot blot results. Dot blot overlay analysis of ORF55 interaction with polypeptide in vitro. **B** Prediction of interactions. Chimera was used to construct 3D models, and Autodock 4.2 was used to perform protein-peptide docking

Through analysis of the phage display results described above, we obtained a polypeptide sequence of 12 amino acids. In this experiment, we used the dot blot method for validation. When the concentration of the ORF55 protein was > 0.5 mg/mL, the dot blot results showed a significant band, with the MBP protein tag alone serving as a negative control (Fig. 4A).

In order to further understand the role of the ICG (ligand) in nanostructure formation, ORF55 was used as a macromolecule and the polypeptide was used as a ligand for in silico analysis of the interaction between them during NP formation. The docking results revealed active binding between the ORF55 protein and the polypeptide. Specifically, binding between the two was due to interactions between a lysine residue of the ORF55 protein and a sulphonate group of the polypeptide (Fig. 4B). It was found that the docked ligand is close to the lysine and lies within hydrogen bonding distance of the sulphonate group. The distance from the lysine residue of PLL to the adjacent oxygen atom of ICG is in the range of 1.8 Å to 2.2 Å. Docking studies revealed that the docked ICG forms two hydrogen bonds with the lysine, with a bond length < 2.2 Å, suggesting possible aggregation of the ICG molecule within PLL/salt aggregates.

### Bioinformatics analysis of potential interacting proteins

**Fig. 5:**
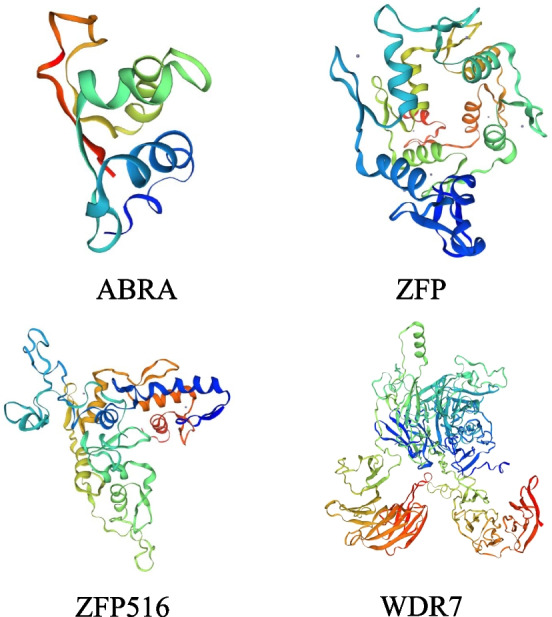
Tertiary structure analysis. Tertiary structures for five potential interacting proteins were predicted

Amino acid sequences were analysed by alignment to the NCBI crucian carp and goldfish databases, and the ORF55 protein was predicted to interact with four proteins (Table 4D). They were gastrula zinc finger protein XlCGF8.2DB-like (ZFP, GenBank accession NO. XP_026094447.1), WD repeat-containing protein 7 isoform X1 (WDR7, XP_026052722.1), actin-binding Rho-activating protein (ABRA, XP_026140919.1) and zinc finger protein 516-like Isoform X1 (ZFP516, XP_026087732.1). A brief analysis of the respective physical and chemical parameters of the above proteins was performed (Additional file 1: Table S1). The Swiss-model (https://swissmodel.expasy.org/) was used to predict the tertiary structures (Fig. 5) and the evolutionary tree (Additional file 2: Fig. 3G) of the four proteins. The results showed that all four shared at least 70% sequence similarity between homologs in different species, indicating high conservation between their respective gene families. The results of conserved domain analysis showed that all four proteins had at least one domain or superfamily structure (Additional file 1: Table S2) potentially playing a specific role in the process of viral infection.

### RT-qPCR verification

**Fig. 6:**
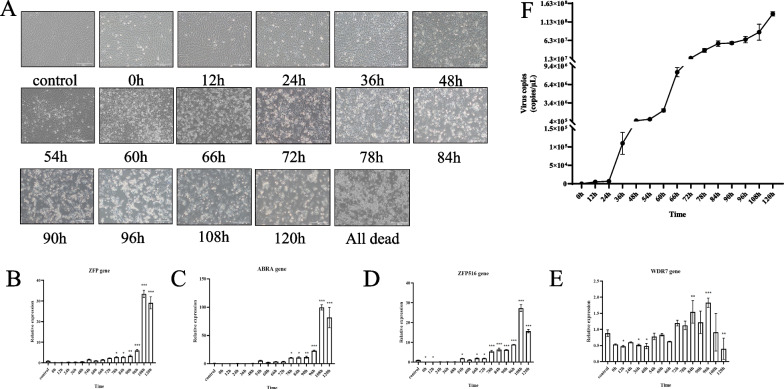
Images of virus-infected cells and RT-qPCR results. **A** Viral infected cells. GiCF cells were infected with the supernatant of strain YC-01 stored in our laboratory, and sampled at different timepoints (0 h, 12 h, 24 h, 36 h, 48 h, 54 h, 60 h, 66 h, 72 h, 78 h, 84 h, 90 h, 96 h, 108 h and 120 h). Over time, CPE of cells gradually became obvious until all cells died. **B**–**E** RT-qPCR results. RNA was extracted by Trizol reagent and reverse-transcribed to cDNA to be used as template. The above genes were quantified using a real-time instrument. Error bars represent standard deviation of the mean for experiments performed in triplicate (*p ≤ 0.05, **p ≤ 0.01, ***p ≤ 0.001). **F** Virus titre analysis. Titre values after viral infection of cells were determined at different timepoints

GiCF cells were infected with the supernatant of CyHV-2 YC-01 and sampled at different timepoints (0 h, 12 h, 24 h, 36 h and 48 h). A significant CPE was observed under the microscope ~ 48 h after viral infection, and all cells had died ~ 144 h after infection (Fig. 6A). According to the nucleic acid sequences published by NCBI, RT-qPCR primers were designed (Table 5). The results showed that the relative expression of ZFP, ABRA and ZFP516 was upregulated in the latter stages of viral infection, without a significant change in WDR7 expression at any of the timepoints (Fig. 6B − E). Melting curves of RT-qPCR experiments are shown in Additional file 2: Fig. 4. Additionally, viral titres were tested, and the results showed a significant increase with increasing time, as predicted (Fig. 6F).

**Table 5:** Oligonucleotide primers and conditions for Real-time PCR

RT-PCR	Gene Name	Nucleotide sequence(5’-3’)	Amplicon size(bp)	Tm(℃)
1	ZFP	F:CCGTTCACCTGCCATCATTG	74	60
		R:ACATGACGGCTCACATGACT		
2	WDR7	F:ATCACTGCAGGCTGTCTGTC	242	60
		R:AGGGCTTTACAGGTGTGTCG		
3	ZFP516	F:TGATGCCATGGCGAGTATCC	152	60
		R:TAGGCCGGTACTCAGTGTCA		
4	ABRA	F:GCACCGTATCCGTCAAAGGT	102	60
		R:GAGTCACCTTGCCATTGCTG		
5	β-actin	F:CACTGTGCCCATCTACGAG	224	55
		R:CCATCTCCTGCTCGAAGTC		

Based on the results of the reciprocal polypeptide screen, complete nucleic acid sequences were searched from NCBI and RT-qPCR primers were designed separately. The β-actin gene refers to the housekeeping gene in the relative quantification

### Subcellular localisation

**Fig. 7:**
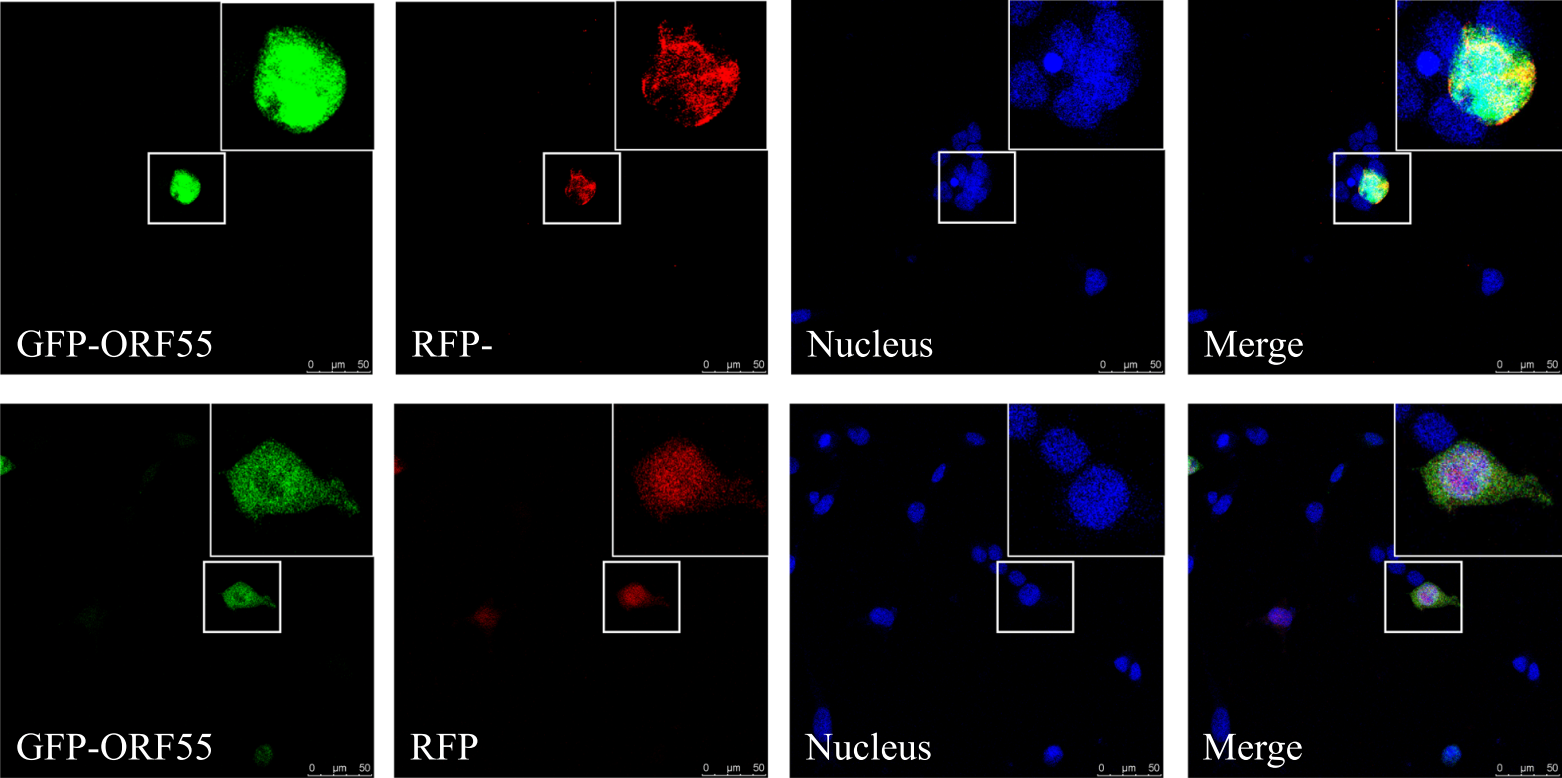
Subcellular localisation. HEK 293 T cells were transfected with pEGFP-N1-ORF55, pDsRed-Express-N1-ZFP and pDsRed-Express-N1-ABRA for 40 h. RFP was used as a negative control

Through the results of the relative expression of four genes described above, we inferred that ABRA might interact with the ORF55 protein, and attempted to validate this. Both pEGFP-N1 and pDsRed-Express-N1 were chosen to connect ORF55 and ABRA genes, using primers listed in Table 6. Both were cotransfected into HEK-293 T cells, and RFP empty vector served as a control. After 40 h, cells were transferred to a confocal microscope, observed, and photographed. The results showed that the ORF55 protein colocalised with ABRA in the nuclei of 293 T cells, but it did not colocalise with RFP, indicating that the RFP tag and ORF55 protein were located in different organelles, respectively. (Fig. 7).

**Table 6:** Primer sequences for Dual-luciferase reporter gene assay

PCR	Luciferase	Gene Name	Nucleotide sequence(5’-3’)	Amplicon size(bp)
1	pEGFP-N1	ORF55	F:GGACTCAGATCTCGAGATGGCTTTTCTGGAGTTGGTGC	645
			R:GGCGACCGGTGGATCCCGTACAAAAGAACAAGGGGCATCCG	
2	pDsRed-Express-N1	ABRA	F:TACCGGACTCAGATCTCGAGATGGAGACAGACGAAGCACC	474
			R:TGGTGGCGACCGGTGGATCCCGCTGTAACAAGGTGATGAGCACT	
3	pEGFP-N1	GFP	F:CGCAAATGGGCGGTAGGCGTG	227
			R:CGTCGCCGTCCAGCTCGACCAG	
4	pDsRed-Express-N1	RFP	F:ATAGCGGTTTGACTCACGGG	585
			R:GCCGTCCTCGAAGTTCATCA	

Based on the sequences of ORF55 and ABRA provided by NCBI, common PCR primers were successfully designed and applied to subsequent ligation of dual-fluorescein reporter vectors. Universal primers used for sequencing were also designed

### Coimmunoprecipitation

**Fig. 8:**
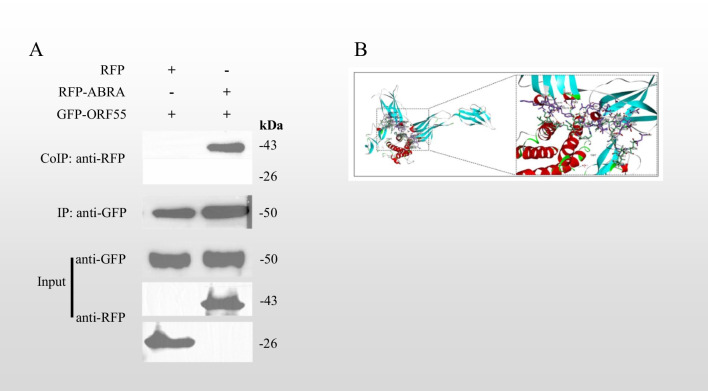
Verification of the interaction between ORF55 protein and ABRA. **A** Molecular docking of ORF55 protein and ABRA. Home for researchers was used to analyse the specific interaction binding sites of the two proteins. **B** Results of coimmunoprecipitation. After taking cell samples for validation analysis, immunoprecipitation magnetic beads were used to purify and separate interacting proteins

Cell samples after successful transfection as described above were analysed by western blotting. The experiment was divided into two parts; the first set of samples contained RFP and GFP-ORF55 protein, and the second set of samples contained RFP-ABRA and GFP-ORF55 protein. Both were recognised by GFP antibodies in the input (IP) group, while the RFP antibody only recognised 26 kDa and 43 kDa proteins, respectively. The IP group confirmed that the GFP magnetic beads had successfully adsorbed the GFP-ORF 55 protein. The final coimmunoprecipitation results revealed a clear band at 43 kDa in the second set of samples, implying an interaction between the GFP-ORF55 protein and the RFP-ABRA protein, but no interaction with the RFP protein (Fig. 8A).

Furthermore, we used molecular docking to verify the interaction between the ORF55 protein and ABRA, and to make specific binding site predictions. ZDOCK Score values and their best pose interactions were calculated (Table 7). PROK2 forms hydrogen bonds with amino acids including ARG167-ASP109 and ARG167-ASP112. Comprehensive analysis revealed that proteins PROK2 and CSF3 formed stable protein docking models (Fig. 8B).

**Table 7:** Results of molecular docking

Receptor	Ligand	ZDOCK socre	Hydrogen bond interaction	Electrostatic interaction
PROK2 (1IMT)	CSF3 (2D9Q)	− 85.085	B:ARG167:NH1-A:ASP109:OD1,	A:LYS16:NZ-B:ASP197:OD1,
			B:ARG167:NH2-A:ASP112:OD1,	A:LYS16:NZ-B:ASP200:OD1,
			B:ARG288:NH2-A:GLU19:OE1,	B:ARG167:NH1-A:ASP112:OD2,
			A:HOH177:O-A:PRO65:O…	B:ARG288:NH1-A:GLU19:OE2,
				A:LEU15-B:LEU291

Rigid protein–protein docking (ZDOCK) was performed between PROK2 and CSF3 to study the relationships. The ZDOCK module was run to identify the docking sites and calculate the ZDOCK scores

## Discussion

Outbreaks of CyHV-2 with a mortality rate of > 90% and no effective treatment measures have been reported in many countries and regions. There are three virus types in the Alloherpesviridae family, among which CyHV-1 is the least harmful, hence it is rarely reported. CyHV-3, also known as Koi herpesvirus (KHV) [36], causes a large number of koi deaths worldwide, hence it has received international attention. There have been some studies on virulence genes of KHV [37], but research on CyHV-2 protein is limited, and the main focus has been on the identification and functions of major membrane proteins and capsid proteins, as well as immunological detection methods [38]. The function and virulence mechanism of the CyHV-2 virulence remain unknown.

In the field of virus research, the *E. coli* prokaryotic expression system has many advantages including relatively low cost and simple operation, hence it is widely employed, as demonstrated for Duck Tembusu virus (DTMUV) non-structural protein 2A (NS2A) [39]. The CyHV-2 protein received some attention [40]. Therefore, we wished to set up a prokaryotic expression system and purify recombinant ORF55 in the present work. According to the preliminary analysis of physicochemical parameters, the ORF55 protein was unstable and not highly hydrophilic (Table 1), indicating that the final purified protein may not be highly soluble and/or easily degraded. Based on these analyses, the pMAL-c5X vector was selected for gene cloning because MBP is known to be highly soluble [41] and can enhance the biological activity of fused target proteins [42]. We successfully cloned the ORF55 gene into the pMAL-c5X vector, and expressed and purified the MBP-ORF55 fusion protein.

We then explored the reference lists of relevant studies on potential interacting proteins (Table 4D). Zinc finger proteins (ZFPs) are a class of proteins that contains short, stable, self-folding ‘finger’ structures that bind zinc ions. Studies have shown that zinc-finger proteins are natural host endogenous immune factors that regulate expression of cytokines such as interleukins and interferons, and directly regulate lymphocyte growth and differentiation [43]. The human specificity protein 1 (SP1) transcription factor belongs to the *C2H2*-type zinc finger family, and has been widely studied in HeLa cells (human cervical cancer cells) [44]. This protein regulates gene expression by interacting with other proteins, acting as a negative or positive regulator of gene expression [45]. In addition, a class of zinc-finger antiviral proteins has been widely studied in land animal viruses, including Small Ruminant Morbillivirus (SRMV) [46], Hepatitis E virus (HEV) [47] and Human immunodeficiency virus [48]. Members of this class of proteins can have specific inhibitory effects on viral replication.

Actin-binding Rho-activating Protein (ABRA, also known as Stars) is a muscle-specific actin-binding protein, a novel and evolutionarily conserved actin-binding protein (ABP) that binds to I-Band in sarcomeres and actin filaments in transfected cells to activate Rho signalling events [49]. ABRA may be involved in regulating actin cytoskeleton functions and actin polymerisation in various cells. Actin is a globular multifunctional protein that forms microfilaments, present in essentially all eukaryotic cells, and is the most abundant cytoskeletal protein in eukaryotic cells. Actin plays a regulatory role in viral infection, and is closely related to virus adsorption and entry, virus replication, virus assembly, virus release, and virus transmission. Studies have shown that actins including Profilins [50], Cofilin [51, 52] and Drebrin [53] can play a key role in viral infection [54]. Rho family proteins were the first cloned proteins in the Ras superfamily; they have GTP hydrolysis activity and play an important role in the regulation of cytoskeletal recombination. Rho is highly expressed in various malignancies and is closely related to tumorigenesis, invasion and metastasis [55].

We also investigated WD repeat-containing protein 7 (WDR7). F-box and WD repeat domain containing 7 (FBXW7) is a member of the F-box protein family that functions as the substrate recognition component of the SCF E3 ubiquitin ligase. FBXW7 is a critical tumour suppressor and is able to control proteasome-mediated degradation of oncoproteins [56]. Herein, the WDR7 gene did not show an obvious change in expression, indicating that it might not be altered during viral infection of cells.

In this experiment, PDT was used to explore a polypeptides interacting with the ORF55 protein, and predict potential interacting host proteins. Putative peptides can then be tested in cells to assess inhibitory or promoting effects on the virus, and further explore its mechanism. Through molecular docking and in vitro experiments, we determined that ABRA could interact with the ORF55 protein. In future work, the mechanism of interaction will be further investigated. For example, RNA interference (RNAi) and overexpression can be used to determine the nature of interactions, and signalling pathways can be studied by transcriptome analysis. For the other three identified interacting proteins, other methods can employed to probe interactions with the ORF55 protein. This screening results provide a basis for exploring the specific biological functions of the ORF55 protein in the mechanism of virus infection.

## Conclusion

In summary, we successfully constructed a prokaryotic expression system for the CyHV-2 YC-01 TK gene, and applied the resulting purified ORF55 protein in phage display experiments. A polypeptide sequence interacting with the ORF55 protein was successfully screened, and four potential interacting proteins were predicted using the NCBI database. Following validation by RT-qPCR and coimmunoprecipitation experiments, we determined that the ORF55 protein could interact with ABRA. The findings lay a foundation for the further investigation of the infection mechanism of ORF55.

## Supplementary Information

**Additional file 1.** Analysis of physical and chemical parameters and conserved domain of interactive protein.

**Additional file 2.** Bioinformatic analysis and melting curves.

## Data Availability

Not applicable.
