# Supplementary material for: Screening and identification of cyprinid herpesvirus 2 (CyHV-2) ORF55-interacting proteins by phage display
Source: Virol J. 2023 Apr 12;20:66. doi: 10.1186/s12985-023-02026-x (PMC10091560; doi:10.1186/s12985-023-02026-x)
Supplement: Supplementary file 1 — Additional file 1. Analysis of physical and chemical parameters and conserved domain of interactive protein. [file 12985_2023_2026_MOESM1_ESM.docx]

**Additional file**

**Table 1. Analysis of physical and chemical parameters of interactive protein**

| **Protein** | **AA** | **MW** | **pI** | **EC** | **Instability Index** | **Aliphatic Index** | **GRAVY** |
| --- | --- | --- | --- | --- | --- | --- | --- |
| ABRA | 157 | 17.67 | 5.67 | 20970 | 31.95 Stable | 70.70 | -0.812 |
| ZFP | 371 | 43.25 | 9.51 | 4470 | 32.61 Stable | 51.46 | -0.836 |
| ZFP516 | 1065 | 118.64 | 8.83 | 86290 | 53.14 Unstable | 54.42 | -0.946 |
| WDR7 | 1466 | 160.82 | 6.54 | 154700 | 51.06 Unstable | 92.14 | -0.071 |

**Table 1.** Analysis of physical and chemical parameters of proteins that may interact with the ORF55 protein. ProtParam was used to predict the molecular weight, theoretical protein isoelectric point (pI), molar extinction coefficient, instability index and aliphatic index of the interactive protein. Actin-binding Rho-activating protein (ABRA, GenBank accession NO. XP_026140919.1), Gastrula zinc finger protein (ZFP, XP_026094447.1), Zinc finger protein 516 (ZFP516, XP_026087732.1), WD repeat-containing protein 7 (WDR7, XP_026052722.1).

**Table 2. The conserved domain of the above 4 proteins**

| **Protein name** | **Conserved domains** | **Accession** | **Position** | **E values** |
| --- | --- | --- | --- | --- |
| ABRA | Costars superfamily | pfam14705 | 81-155 | 1.48e-41 |
| ZFP | zinc-finger double domain | pfam13465 | 138-163 | 1.30e-03 |
|  | COG5048 superfamily | COG5048 | 207-357 | 3.92e-05 |
| ZFP516 | zinc-finger double domain | pfam13465 | 41-66 | 2.61e-05 |
|  |  |  | 999-1024 | 1.34e-04 |
|  | Zinc finger (C2H2 type) domain | pfam00096 | 27-49 | 7.22e-03 |
|  |  |  | 985-1007 | 4.36e-04 |
| WDR7 | WD40 superfamily | cd00200 | 22-214 | 6.14e-10 |
|  |  |  | 458-600 | 1.09e-15 |
|  |  |  | 1333-1402 | 5.56e-09 |

**Table 2.** Conserved domain analysis. The results of conserved domain analysis showed that all four proteins had at least one domain or superfamily structure, potentially playing a specific role in the process of viral infection.
