# Supplementary material for: Screening and identification of cyprinid herpesvirus 2 (CyHV-2) ORF55-interacting proteins by phage display
Source: Virol J. 2023 Apr 12;20:66. doi: 10.1186/s12985-023-02026-x (PMC10091560; doi:10.1186/s12985-023-02026-x)
Supplement: Supplementary file 2 — Additional file 2. Bioinformatic analysis and melting curves. [file 12985_2023_2026_MOESM2_ESM.ppt]

## Slide 1
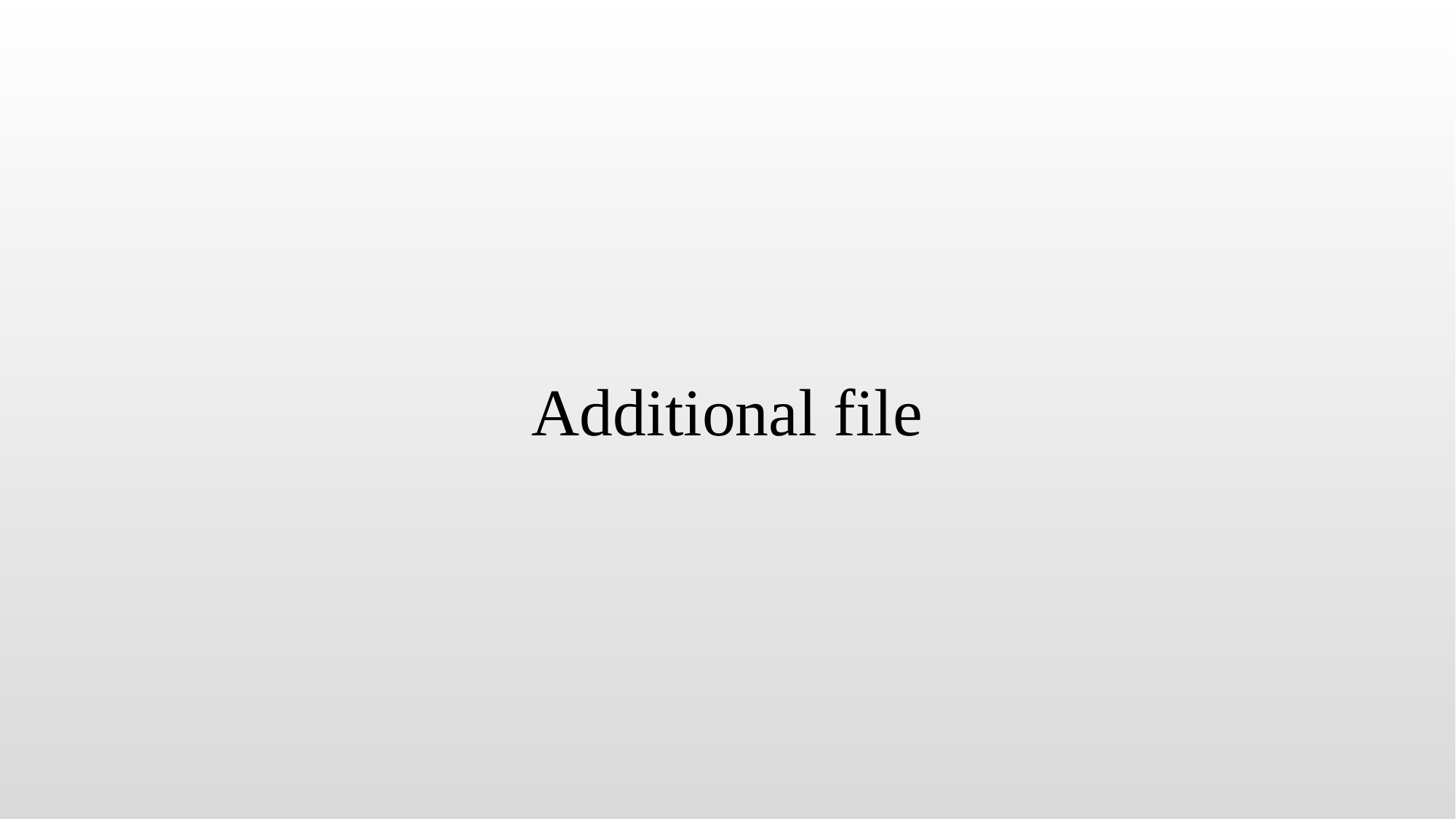

Additional file

## Slide 2
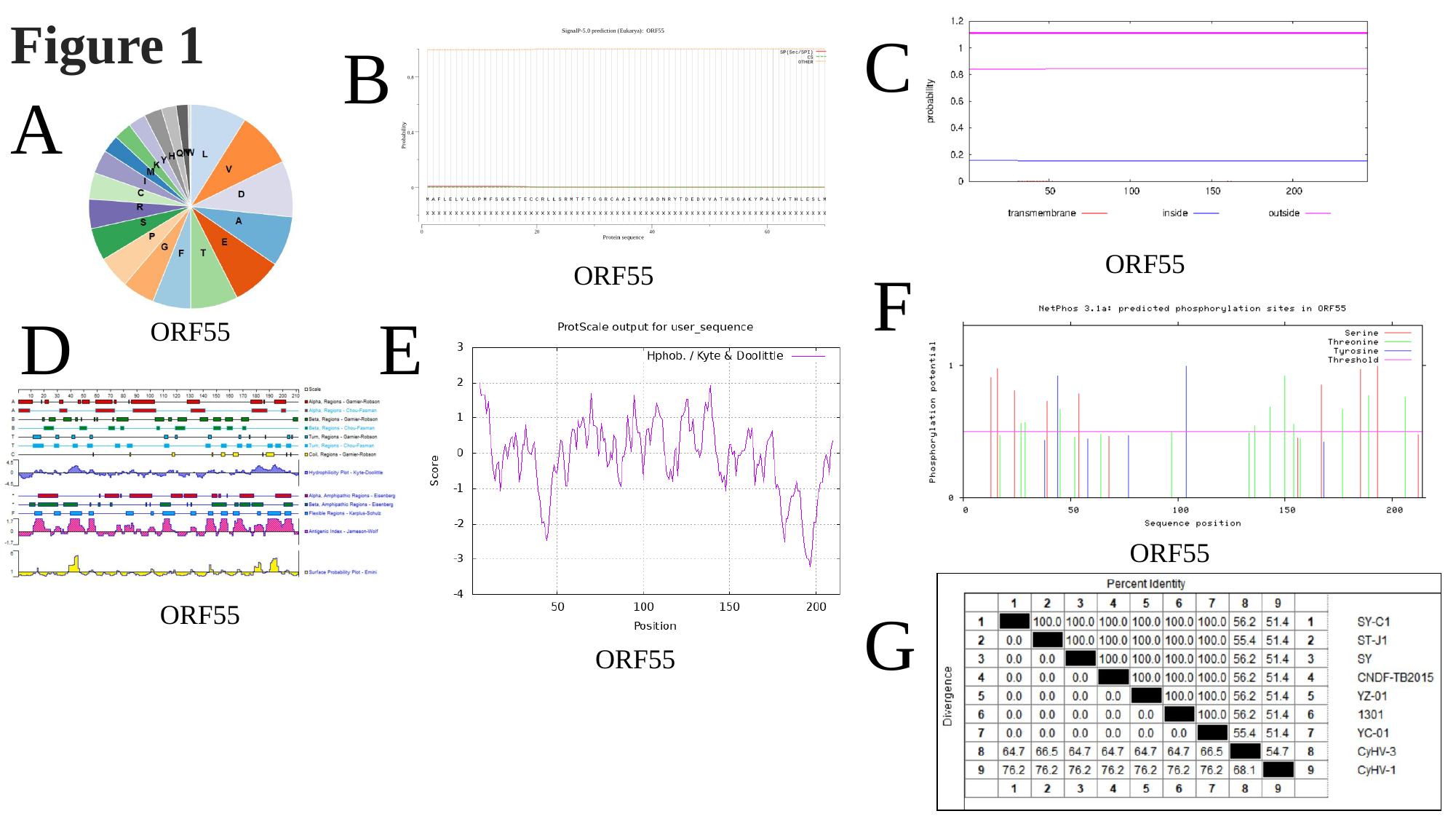

# Figure 1
C
B
A
ORF55
ORF55
F
D
E
ORF55
ORF55
G
ORF55
ORF55

## Slide 3
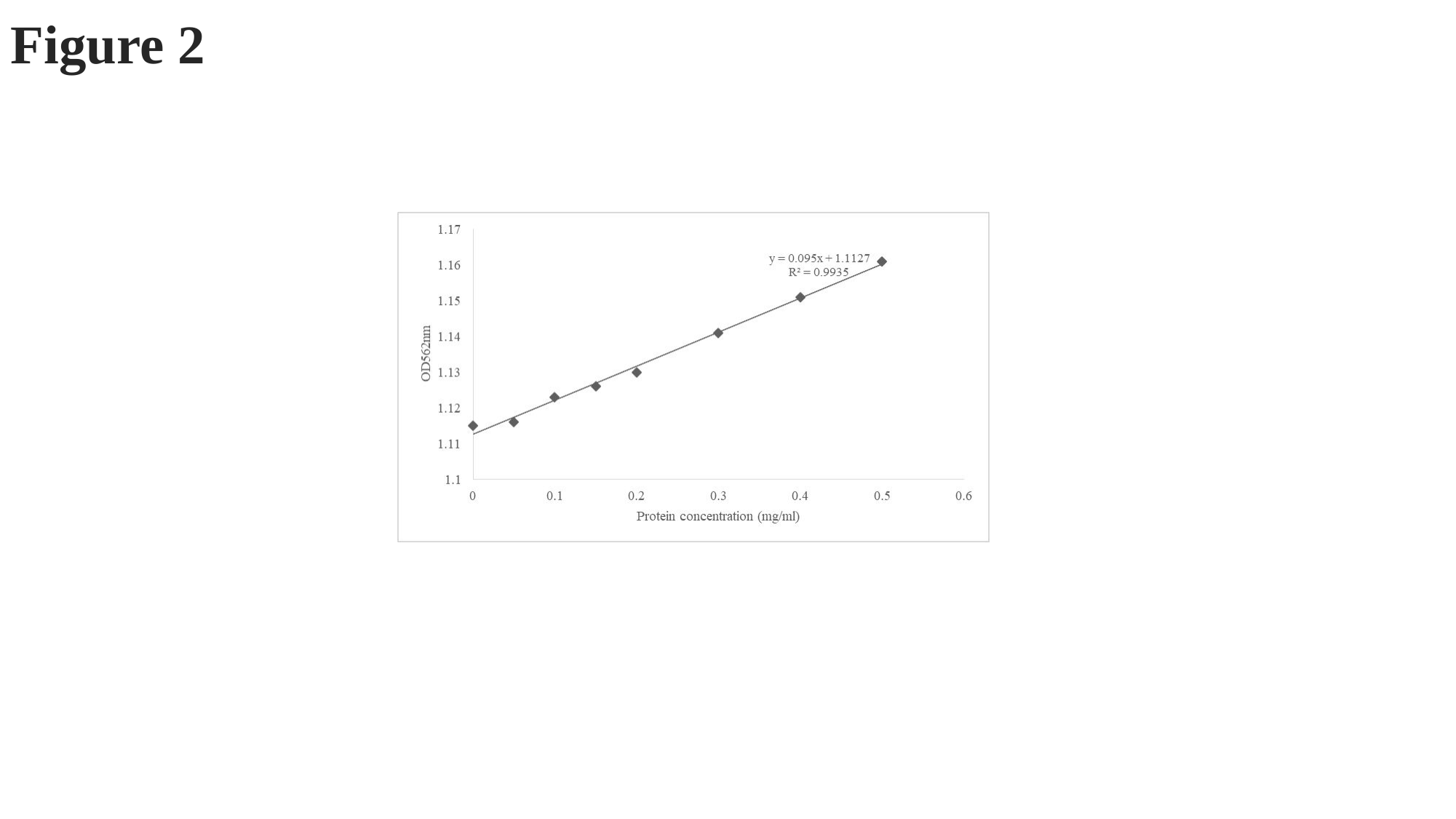

Figure 2

## Slide 4
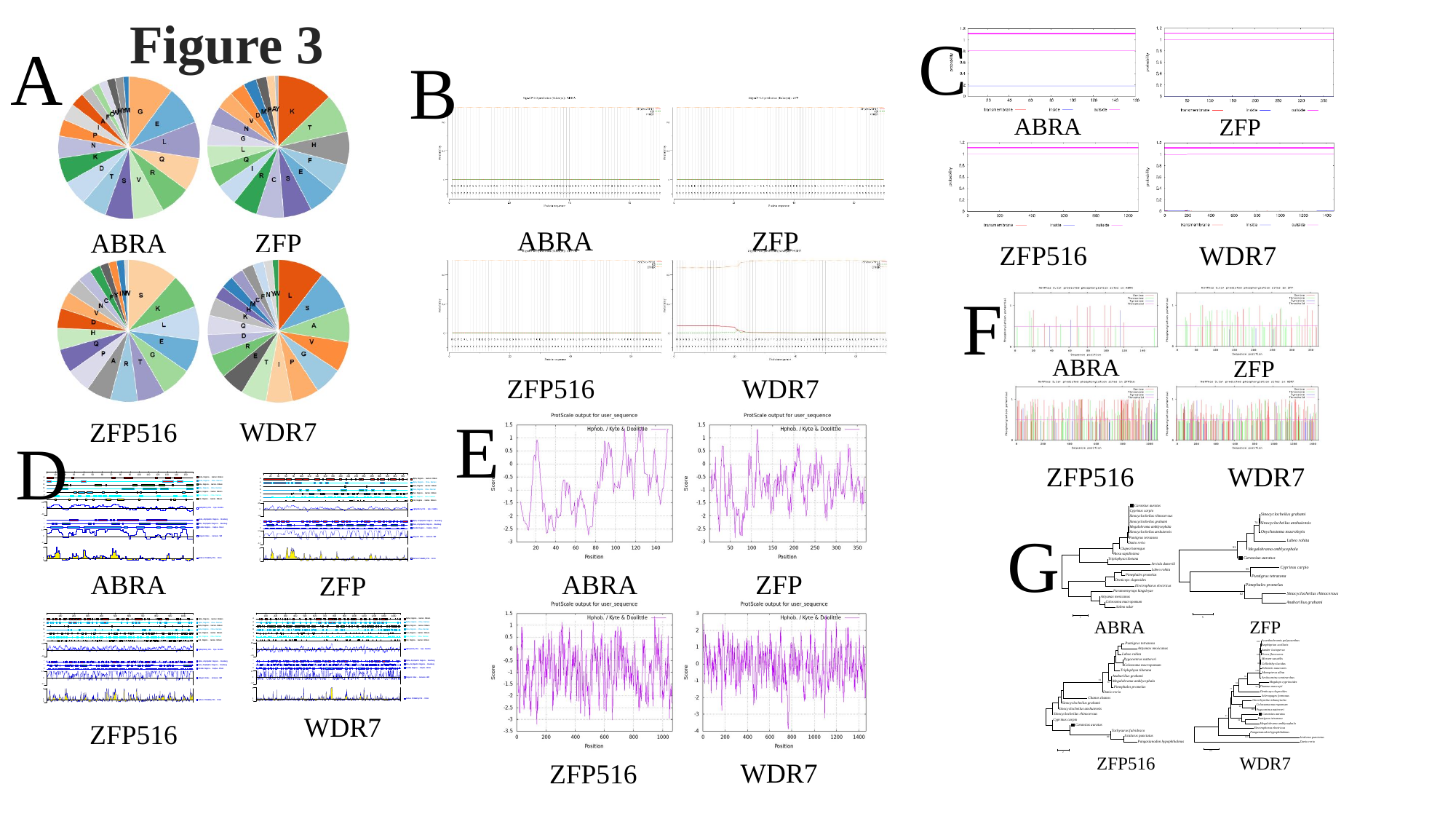

# Figure 3
C
ABRA
ZFP
ZFP516
WDR7
A
ABRA
ZFP
WDR7
ZFP516
B
ABRA
ZFP
ZFP516
WDR7
F
ABRA
ZFP
ZFP516
WDR7
E
ABRA
ZFP
WDR7
ZFP516
D
ABRA
ZFP
ZFP516
WDR7
G
ABRA
ZFP
WDR7
ZFP516

## Slide 5
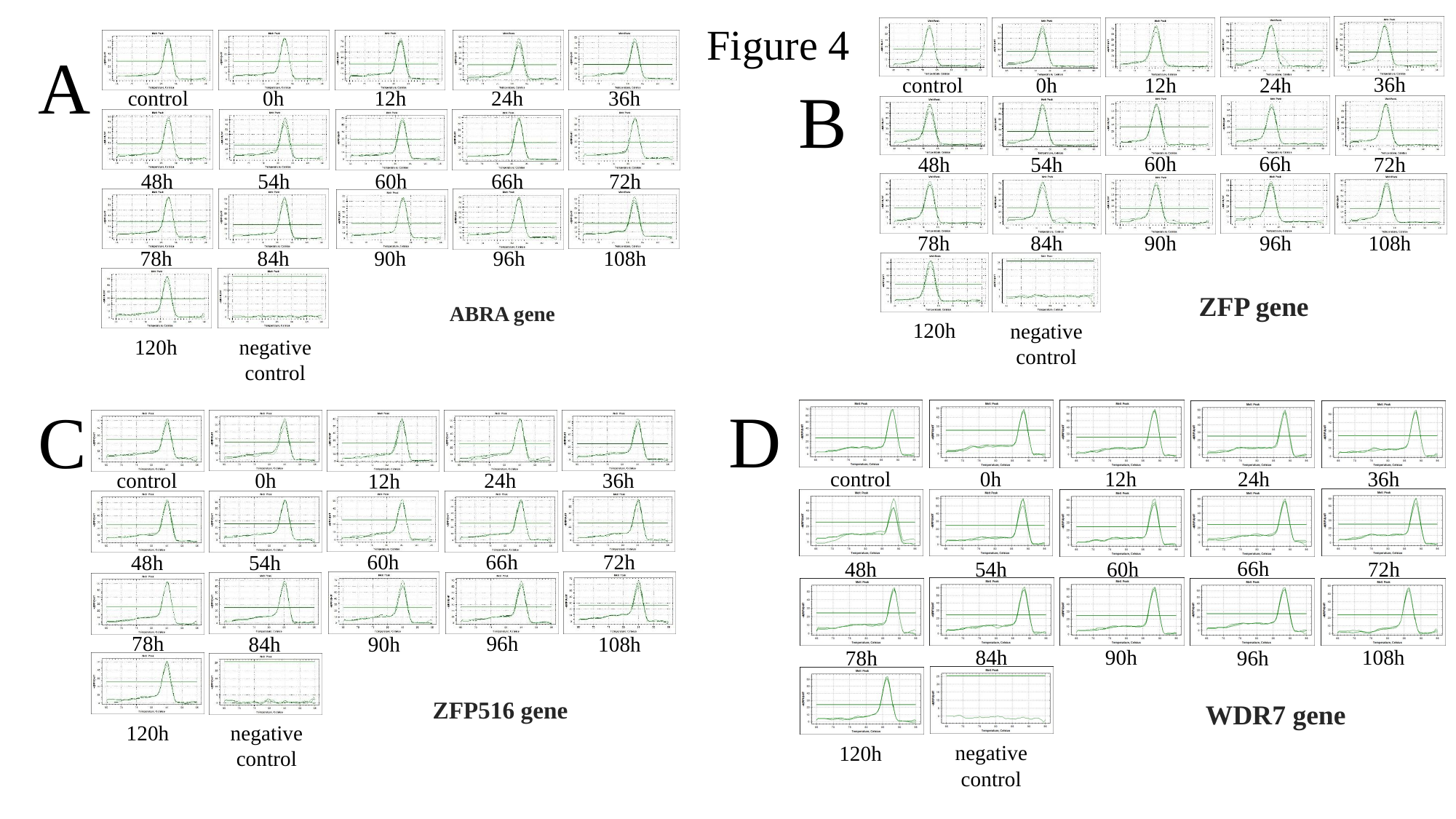

Figure 4
36h
0h
control
24h
12h
60h
66h
72h
48h
54h
96h
108h
78h
84h
90h
120h
negative control
control
0h
12h
24h
36h
48h
60h
72h
54h
66h
78h
90h
108h
84h
96h
120h
negative control
A
B
ZFP gene
# ABRA gene
D
C
control
0h
12h
36h
24h
66h
54h
60h
72h
48h
108h
84h
90h
78h
96h
negative control
120h
0h
36h
control
24h
12h
72h
60h
66h
54h
48h
96h
78h
90h
108h
84h
negative control
120h
ZFP516 gene
WDR7 gene
